# Prevalence of *Toxoplasma gondii* infection in animals of the Arabian Peninsula between 2000–2020: A systematic review and meta‐analysis

**DOI:** 10.1002/vms3.1004

**Published:** 2022-11-21

**Authors:** Asmaa Abdelgadier, Nada Assaad, Zaynab Elhussein, Abdulla M. Al‐Marri, Sami Suliman, Khalid Eltom, Ebtisam A. Al‐Mslemani, Abdul Azia Al‐Zeyara, Abdel Rahim M. El Hussein, Khalid A. Enan

**Affiliations:** ^1^ Department of Biological and Environmental Sciences College of Arts and Sciences Qatar University Doha Qatar; ^2^ Department of Veterinary Laboratory Animal Resources Ministry of Municipality Doha Qatar; ^3^ Medical Laboratory Sciences Ibn Sina University Khartoum Sudan; ^4^ Department of Virology The Central Laboratory Ministry of Higher Education and Scientific Research Khartoum Sudan; ^5^ Department of Epidemiology and Infectious Diseases Animal Resources Ministry of Municipality Doha Qatar; ^6^ Animal Resources Ministry of Municipality Doha Qatar

**Keywords:** animals, Arabian Peninsula, meta‐analysis, *Toxoplasma gondii*, toxoplasmosis

## Abstract

**Background:**

*Toxoplasma gondii* (*T. gondii*) is a zoonotic parasite that can be transmitted from animals to humans, with felids acting as its definitive host. Thus, understanding the epidemiology of this parasite in animal populations is vital to controlling its transmission to humans as well as to other animal groups.

**Objectives:**

This systematic review and meta‐analysis aims to summarise and analyse reports of *T. gondii* infection in animal species residing in the Arabian Peninsula.

**Methods:**

: It was conducted in accordance with the Preferred Reporting Items for Systematic Reviews and Meta‐Analyses (PRISMA), with relevant studies being retrieved from MEDLINE/PubMed, Scopus, Cochrane Library, Google Scholar and ScienceDirect. All articles published in Arabic or English languages between January 2000 and December 2020 were screened for eligibility. Random effects model was used to calculate the pooled prevalence of *T. gondii* infection in different animal populations which were found to harbour this infection. The critical appraisal tool for prevalence studies designed by the Joanna Briggs Institute (JBI) was used to assess the risk of bias in all included studies.

**Results:**

A total of 15 studies were retrieved, reporting prevalence estimates from 4 countries in this region and in 13 animal species. Quantitative meta‐analysis estimated a pooled prevalence of 43% in felids [95% confidence interval (CI) = 23–64%, *I* ^2^ index = 100%], 48% in sheep (95% CI = 27–70%, *I* ^2^ = 99%) and 21% in camels (95% CI = 7–35%, *I* ^2^ = 99%). Evidence of possible publication bias was found in both felids and sheep.

**Conclusions:**

This meta‐analysis estimates a high prevalence of *T. gondii* infection in animal species which are of high economic and cultural importance to countries of this region. Hence, these findings provide valuable insight to public health authorities as well as economic and animal resources advisors in countries of the Arabian Peninsula.

## Graphical abstract

*Toxoplasma gondii* (*T. gondii*) is a zoonotic parasite that can be transmitted from animals to humans, with felids acting as its definitive host. Thus, understanding the epidemiology of this parasite in animal populations is vital to controlling its transmission to humans as well as to other animal groups. This systematic review and meta‐analysis aimed to summarize and analyze reports of *T. gondii* infection in animal species residing in the Arabian Peninsula.

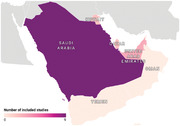

## 1 INTRODUCTION

*Toxoplasma gondii* (*T. gondii*) is an intracellular parasite, and the causative agent of toxoplasmosis which manifests primarily in warm‐blooded species such as felids, birds, humans, and other mammals (Furtado et al., 2011). *T. gondii* has a worldwide distribution, with felids (*Felidae)* acting as its definitive hosts, and various other animal species (including humans) acting as its intermediate hosts. These protozoa can access the host's body through the ingestion of food, water, or coming into contact with soil that is contaminated with *T. gondii* oocyst‐positive felid faeces (Hill & Dubey, 2002). Transmission can also take place through the consumption of undercooked meat or marine molluscs that are contaminated with tachyzoites, bradyzoites or oocysts (Hill & Dubey, 2002). Furthermore, less common routes of *T. gondii* transmission have been reported in humans such as through blood transfusions, organ transplantations and mother‐to‐child transmission (Smith et al., 2021).

Toxoplasma‐infected immunocompetent felids are usually asymptomatic while shedding *T. gondii* oocysts in their faeces (Gastón, 2021). However, in very young or immunocompromised felid hosts, tachyzoites can disseminate systemically causing interstitial pneumonia, myocarditis or hepatic necrosis (Matta et al., 2021). Additionally, *T. gondii* infection is among the most important causes of abortion and stillbirth in sheep, goats, cervids and pigs; the mechanism of which have been previously described in ewes by Gastón (2021), as blood‐borne tachyzoites were found to infect placental cotyledons resulting in their necrosis (Gastón, 2021).

*Toxoplasma* infection is usually sub‐clinical in immunocompetent humans. However, any deterioration of the immune system's activity significantly increases the risk of *T. gondii* reactivation, and the development of possibly fatal clinical manifestations such as cerebral toxoplasmosis (Wang et al., 2017). Furthermore, women of childbearing age that acquire the infection during the last three months before conception, run a high risk of the mother‐to‐child parasite transmission during pregnancy that results in the development of congenital toxoplasmosis in the newborns (Chaudhry et al., 2014). If not properly treated, infected newborns can develop serious post‐natal manifestations such a retinochoroiditis, and neurological deficits either in childhood or early adulthood (Chaudhry et al., 2014).

This systematic review and meta‐analysis aims to describe the prevalence of *T. gondii* in the animal populations of the Arabian Peninsula, a region where people live and work in close proximity to animals, which are possible carriers of *T. gondii*, and where animals like sheep, camels and goats are usually slaughtered in millions per annum as part of local cultural celebrations or religious ceremonies.

## 2 METHODS

This systematic review and meta‐analysis was conducted in full accordance with the Preferred Reporting Items for Systematic Reviews and Meta‐Analyses (PRISMA) (Moher et al., 2009) (Supplementary File 1). The review protocol was registered in Open Science Framework under the DOI: 10.17605/OSF.IO/EYZDW.

### 2.1 Eligibility criteria

Countries of the Arabian Peninsula include all of the following: Bahrain, Kuwait, Oman, Qatar, Saudi Arabia, United Arab Emirates and Yemen. We considered all studies written either in Arabic or English languages. We limited our review to studies which were published between January 2000 and December 2020 in order to provide a contemporaneous overview of toxoplasmosis epidemiology in this region. Eligible studies were those which reported a prevalence estimate of toxoplasmosis in at least one animal species. On the other hand, the following exclusion criteria were applied: (i) studies of toxoplasmosis in humans; (ii) reviews, editorials, letters to the editor, commentaries, conference proceedings, case reports and case series; and (iii) studies which investigated toxoplasmosis in samples which had been collected prior to the eligible study period of our review. No exclusion was done based on breeding environments, study setting or the used diagnostic techniques.

### 2.2 Search strategy

Comprehensive literature search was commenced in December 2020. The following databases were screened for eligible studies: MEDLINE/PubMed, Scopus, Cochrane Library, Google Scholar and ScienceDirect. These databases were queried to search the ‘Titles and Abstracts’ of articles. Keywords used in this search were different combinations of the words ‘*toxoplasma*’, ‘toxoplasmosis’ and ‘*T. gondii*’ and the names of all eligible countries. When possible, a publication date filter was set to the period between January 2000 and December 2020. Otherwise, manual application of this inclusion criterion was performed in later stages. The search strategy used in MEDLINE/PubMed is shown in Supplementary File 2. Adapted versions of this search strategy were used in all other databases.

**Impact**

- Although felids act as definitive hosts for toxoplasmosis, this infection can be transmitted to humans as well as other animal species (i.e. its intermediate hosts) with devastating consequences.
- Understanding the epidemiology of toxoplasmosis in this region and identifying the at‐risk animal species will guide infection control strategies in breeding centres and veterinary practices.
- Reporting this high prevalence of toxoplasmosis in livestock entices public health authorities to promote safe practice guidelines regarding animal handling, slaughtering techniques and meat processing, as well as providing evidence‐based health education to the public, in order to minimise the spread of this infection to humans.

### 2.3 Study selection

All studies which were retrieved from database search were imported into Mendeley Reference Manager. The same software was used to automatically screen for duplicates which were then excluded. These citations were then transferred to Rayyan software (https://rayyan.qcri.org/) which was used to complete all remaining stages of study selection. Firstly, four reviewers blindly examined all citations in Rayyan to manually exclude any remaining duplicates. Then, titles and abstracts of remaining articles were cross‐examined against our eligibility criteria by four reviewers, working in blinded, independent pairs. Thus, each article was screened for eligibility by two reviewers, and it was only included if the decision was mutual. Any discrepancies were decided upon by two senior reviewers. The full texts of included studies were then retrieved and screened to confirm their eligibility by four reviewers who also worked in blinded, independent pairs. However, each pair was assigned mostly to studies which they had not already evaluated for eligibility in the prior stage. Moreover, corresponding authors were contacted to request full texts of studies which were either not available or inaccessible to the reviewing team. A waiting period of two weeks was decided, and failure to receive a response from an author during this period meant the exclusion of the corresponding study from this review. Grey literature and non‐peer‐reviewed articles were not included in order to maximise the accuracy of the meta‐analysis generated pooled estimates.

### 2.4 Data extraction

Four reviewers worked independently to extract all necessary information from eligible studies. The following variables were extracted from each article: authors, publication date, time of data and samples collection, country and city (or region) of the study, study setting, studied animal species, diagnostic techniques, sample size and prevalence estimates. All variables were entered to, and managed by Microsoft Office Excel 2010. A senior reviewer validated all obtained data by performing a second round of data extraction. The taxonomy of studied animal species was verified and updated using online databases such as the National Center for Biotechnology Information (NCBI) Taxonomy Browser, the Global Biodiversity Information Facility (GBIF), and Animal Diversity Web (ADW) (Supplementary File 3).

### 2.5 Quality assessment

Four reviewers assessed the risk of bias in all included studies using a modified version of the Critical Appraisal Tool for prevalence studies which was designed by the Joanna Briggs Institute (JBI) in 2017 (Munn et al., 2020). This tool was designed to evaluate the risk of selection bias, confounding bias and bias related to measurement and data analysis. It is a 9‐question model, with each question being answered either with a ‘yes’, ‘no’, ‘unclear’ or ‘not applicable’. For each study, a score was calculated as the total number of questions answered with ‘yes’. Studies were then categorised into three groups according to this score as follows: high risk of bias (a score of 0–3), intermediate risk of bias (a score of 4–6) and low risk of bias (a score of 7–9) (Supplementary File 4).

### 2.6 Data analysis

Prevalence estimates were used to conduct a quantitative meta‐analysis to calculate the pooled prevalence of toxoplasmosis, to assess the level of heterogeneity, and to investigate for possible publication bias in all animal species or subgroups. Meta‐analysis of a subgroup was performed only if we were able to retrieve a minimum of 3 independent observations representing that subgroup. If a study reported multiple prevalence estimates (e.g. from different areas or in different animal species), all estimates were entered separately in the meta‐analysis. In each study, the prevalence estimate was used as the effect estimate, while the standard error (SE) was calculated using the following equation:

$$SE=\sqrt{p(1-p)/n},$$

where *p* was the reported prevalence estimate and *n* was the sample size. Studies were weighted according to the effect size and the inverse of variance. We then applied random‐effects model to generate forest plots displaying summary prevalence data with 95% confidence intervals. Moreover, heterogeneity analysis was performed using the Inconsistency Index (*I* ^2^) as it is known to be less influenced by the small number of included studies. A study has low level of heterogeneity if *I* ^2^ is less than 25%, intermediate heterogeneity if *I* ^2^ is 25%–75%, and high heterogeneity if *I* ^2^ is higher than 75%. Finally, funnel plots were generated and visually examined to evaluate for possible publication bias. All of the aforementioned statistical analysis was performed using Review Manager version 5.3 (The Cochrane Collaboration, Copenhagen, Denmark).

## 3 RESULTS

### 3.1 Study characteristics

**FIGURE 1:**
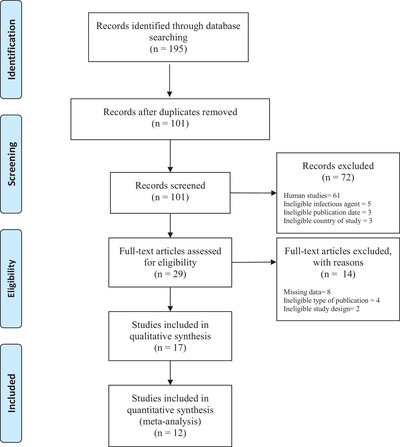
PRISMA (2009) flow chart outlining all stages of study selection.

**FIGURE 2:**
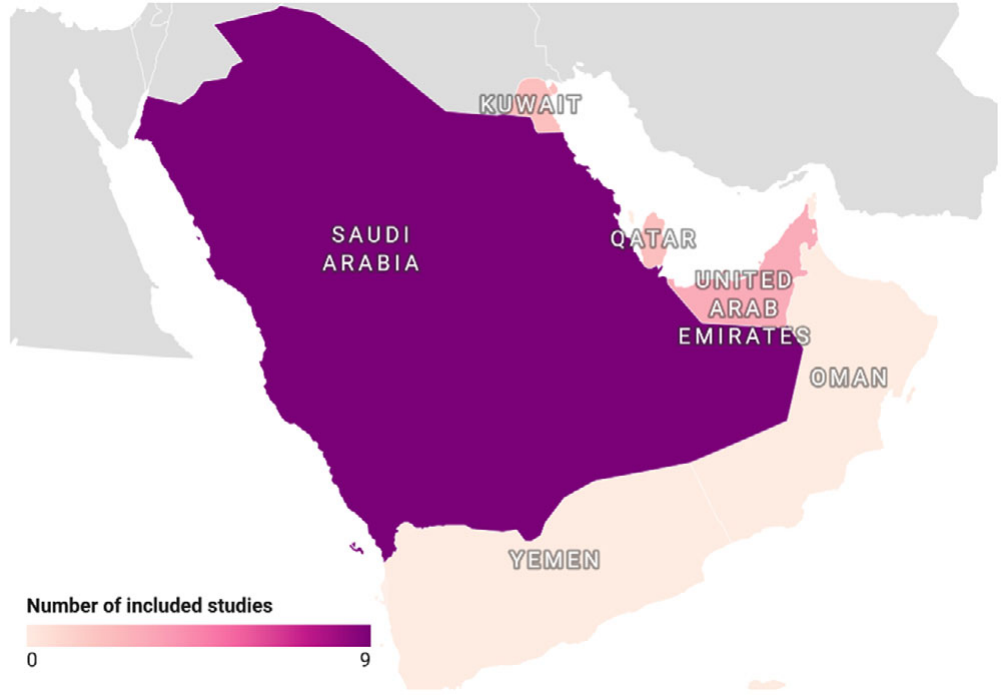
Choropleth map of the Arabian Peninsula illustrating the geographic distribution of included studies

Literature search resulted in the retrieval of 195 studies in total from electronic databases, of which 16 studies fulfilled all eligibility criteria and were included in this review (Figure 1). These studies reported prevalence estimates from 4 countries in the Arabian Peninsula which were: Saudi Arabia (*n* = 9), United Arab Emirates (*n* = 4), Kuwait (*n* = 2) and Qatar (*n* = 2) (Figure 2). Furthermore, in these studies, the following animal sub‐populations were studied: felids (*n* = 6), camels (*n* = 3), sheep (*n* = 3), foxes (*n* = 2), chickens (*n* = 1), goats (*n* = 2), hedgehog (*n* = 1), horses (*n* = 1), jackal (*n* = 1), mongoose (*n* = 1), rats (*n* = 1), hyenas (*n* = 1) and wolves (*n* = 1) (Supplementary File 3). A detailed description of extracted data from included articles is provided in Table 1.

**TABLE 1:** Data extracted from included studies reporting prevalence of *Toxoplasma gondii* in countries of the Arabian Peninsula

First author	Publication date	Years of work	Area	Setting	Studied species	Diagnostic test	Sample size	Prevalence
Saudi Arabia								
**Al‐anazi**	2011	2010	Riyadh	N.S.	Camels	Indirect fluorescent antibody test	412	6.5%
**Alanazi**	2013	2012–2013	Riyadh	governmental slaughter‐houses	Camels	Indirect fluorescent antibody test	1628	23.6%
	Same	Same	Same	Same	Goat	Same	1628	35.3%
	Same	Same	Same	same	Sheep	Same	1628	36.4%
**Alanazi**	2011	N.S.	Riyadh ‐ Al‐Janadriah	Farm	Horses	Standard Sabin‐Feldman dye test	93	33.3%
	Same	Same	Same	Same	Same	Same	35	20.0%
	Same	Same	same	Same	Same	Same	82	27.4%
	Same	Same	Same	Same	Same	Same	21	38%
	Same	Same	Same	Same	Same	Same	27	11.0%
	Same	Same	Same	Same	Same	Same	16	43.7%
	Same	Same	Same	Same	Same	Same	19	26.3%
**Mohammed**	2020	N.S.	Riyadh, Hofuf, Tabuk, Jizan, Taif	Field	Camels	ELISA	199	34.2%
**Nasr**	2018	2013	Qassim	Center of the Arabian Peninsula	Chicken	Direct agglutination test	244	11.9%
	Same	Same	Same	Same	same	PCR	29	100%
	Same	Same	Same	Same	same	Histopathological examination	4	100%
**Mohammed**	2019	N.S.	Riyadh	Field	Felids	ELISA	100	39.0%
	Same	Same	Same	Private pet clinic	Same	ELISA	100	13.0%
**Ismael**	2016	N.S.	Riyadh	Farm	Goat	ELISA	357	15.4%
**Elamin**	2014	N.S.	Riyadh	Collected from different regions in Riyadh	Rats	ELISA	200	13.5%
**Hussein**	2011	2010	Riyadh	Farm A	Sheep	ELISA	168	88.8%
	Same	Same	Same	Same	Same	Indirect haemagglutination test	168	92.3%
	Same	Same	Same	Same	Same	ELISA	52	80.8%
	Same	Same	Same	Same	Same	Indirect haemagglutination test	52	84.6%
	Same	Same	Same	Farm B (King Saud University farm)	Same	ELISA	60	3.3%
	Same	Same	Same	Same	Same	Indirect haemagglutination test	60	3.3%
	Same	Same	Same	Same	Same	ELISA	11	45.4%
	Same	Same	Same	Same	Same	Indirect haemagglutination test	11	36.4%
	Same	Same	Same	Farm A	Same	Histopathological examination	5	40.0%
United Arab Emirates								
**Dubey**	2010	2008–2010	Sharjah	Breeding Centre for Endangered Arabian Wildlife	Felids	Modified agglutination test (MAT)	57	86.0%
			Same	Same	Fox	Same	32	62.5%
			Same	Same	Hedgehog	Same	10	.0%
			Same	Same	Wolf	Same	8	62.5%
			Same	Same	Jackal	Same	8	75%
			Same	Same	Stripped Hyena	Same	6	50.0%
			Same	Same	Mongoose	Same	3	100%
**Dubey**	2008	2006	Sharjah	Breeding Centre for Endangered Arabian Wildlife	Fox	Modified agglutination test (MAT)	12	58.3%
	Same	Same	Same	Same	Same	latex agglutination test (LAT)	12	33.3%
**Pas**	2008	2001,2003–2008	N.S.	Field	Felids	Modified agglutination test (MAT)	36	80.6%
**Schuster**	2009	2004–2008	Dubai	Field	Felids	Faecal sedimentation method	240	0.8%
Kuwait								
**Alazemi**	2014	N.S.	N.S.	N.S.	Sheep	Indirect haemagglutination test	528	17.8%
**Abdou**	2013	2011–2012	N.S.	Trapped from Different areas of Kuwait State	Felids	Indirect haemagglutination test	240	19.6%
	Same	Same	Same	Same	Same	Direct faecal wet mount smears	240	2.1%
Qatar								
**Dubey**	2010	2008–2010	Doha	Al Wabra Wildlife Preservation	Felids	Modified agglutination test (MAT)	21	71.4%
	Same	Same	Same	Same	Same	Same	6	100%
**Boughattas**	2017	2014–2015	Multiple cities in Qatar	Field	Felids	ELISA	495	82.0%

Abbreviations: ELISA: enzyme‐linked immunosorbent assay; N.S.: not specified; PCR: polymerase chain reaction.

### 3.2 Prevalence of toxoplasmosis in felids

**FIGURE 3:**
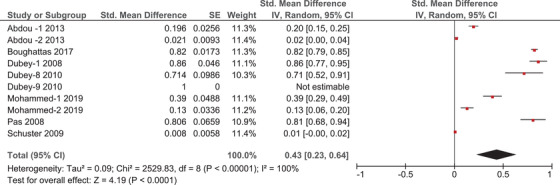
Forrest plot outlining the quantitative meta‐analysis of prevalence estimates of *Toxoplasma gondii* in felids. The central red square represents point estimates, whereas the square size represents the weight of each study in the meta‐analysis.

A total of seven articles reported prevalence estimates of *T. gondii* among several felid species in the Arabian Peninsula. Supplementary File 3 highlights all felid species which were found to harbour *T. gondii* infection in this region. Figure 3 is a forest plot which displays the results of quantitative meta‐analysis in this group. In the forest plots the red square represents the point prevalence estimate in each study, the size of the red square represents the weight of the study in the meta‐analysis, and the horizontal line represents the calculated standard error (SE). In addition, the centre of the diamond sign indicates the pooled prevalence as calculated by meta‐analysis, while the horizontal range of this sign indicates the 95% confidence interval. Prevalence estimates in the forest plots are indicated in decimals (i.e. ranging from 0 to 1). Quantitative meta‐analysis of this subgroup estimated a pooled prevalence of 43% [95% confidence interval (CI) = 23–64%, inconsistency index (*I* ^2^) = 100%] (Figure 3). The high value of *I* ^2^ indicates high heterogeneity between the results of different studies. Furthermore, funnel plot analysis reveals a possible publication bias in this subgroup (Supplementary File 5).

### 3.3 Prevalence of toxoplasmosis in sheep

**FIGURE 4:**
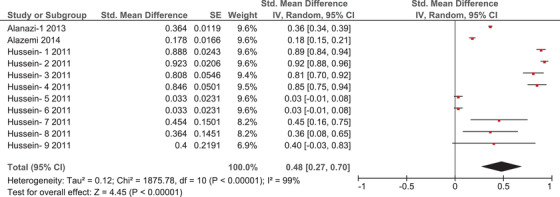
Forrest plot outlining the quantitative meta‐analysis of prevalence estimates of *Toxoplasma gondii* in sheep. The central red square represents point estimates, whereas the square size represents the weight of each study in the meta‐analysis.

Three articles investigated *T. gondii* infections among sheep in this region, all of which were conducted in Saudi Arabia. Those articles reported 11 separate prevalence estimates, and meta‐analysis of these estimates calculated a pooled prevalence of 48% (95% CI = 27–70%, *I* ^2^ = 99%) (Figure 4). The high value of *I* ^2^ indicates high heterogeneity between the results of different studies. Moreover, funnel plot analysis also suggests potential publication bias in this population (Supplementary File 6).

### 3.4 Prevalence of toxoplasmosis in camels

**FIGURE 5:**
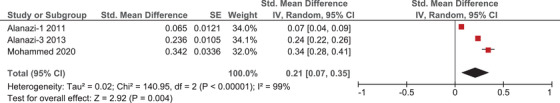
Forrest plot outlining the quantitative meta‐analysis of prevalence estimates of *Toxoplasma gondii* in felids. The central red square represents point estimates, whereas the square size represents the weight of each study in the meta‐analysis.

Three articles reported a total of 3 prevalence estimates of *T. gondii* among camels in the Arabian Peninsula (Table 1). Quantitative meta‐analysis of this subgroup estimates a pooled prevalence of 21% (95% CI = 7–35%, *I* ^2^ = 99%) (Figure 5). The high value of *I* ^2^ indicates high heterogeneity between the results of different studies. Unfortunately, the generated funnel plots for this meta‐analysis were uninterpretable because of the low number of prevalence estimates. Thus, no conclusions on the risk of publication bias in this subgroup could be drawn (Supplementary File 7).

## 4 DISCUSSION

Our meta‐analysis estimates a high prevalence of *T. gondii* among studied animals in the Arabian Peninsula when compared to reports from other regions. Firstly, we estimate a prevalence of *T. gondii* among felids of 43% in this region (Figure 3). This is considerably higher than similar reports from China (24.5%) and Spain (25.5% in pet cats and 36.9% in stray cats) (Ding et al., 2017; Miró et al., 2004). However, even higher estimates have been reported in other countries such as Ethiopia (87.72%) and Estonia (60.8%) (Gebremedhin & Tadesse, 2015; Must et al., 2015). The difference in environmental conditions between these regions is pivotal to explaining this huge variation in prevalence estimates, as feline infections were found to be more common in warm, moist and low‐altitude areas, as these environments seem to facilitate the survival and sporulation of *T. gondii* oocysts (Hatam‐Nahavandi et al., 2021). A similar correlation was also reported in pigs, as higher infection rates can also be seen in regions of low geographical latitudes and high mean annual temperatures (Hatam‐Nahavandi et al., 2021). However, our results show severe heterogeneity between prevalence estimates in felids in the Arabian Peninsula. Methodological diversity can offer one explanation to this high level of heterogeneity, as different studies applied different diagnostic techniques to detect *T. gondii* infection in felids such as enzyme‐linked immunosorbent assay (ELISA), modified agglutination test (MAT), indirect haemagglutination test (IHT) and direct faecal wet mount smears (MW). Ding et al. found a similarly high level of heterogeneity in their meta‐analysis of prevalence reports among felids in China, and they also proposed that methodological diversity might have been a contributing factor to this finding (Ding, 2017). Moreover, clinical diversity is another possible source of this heterogeneity, as these studies report prevalence estimates among felid populations from different countries, with their samples being obtained from various preservations or breeding centres, taking into account that uncontrolled diet, access to contaminated food and water sources, and wandering outdoors can all contribute to a high prevalence of *T. gondii* in felids (Hatam‐Nahavandi et al., 2021). The age of sampled felids is also an important factor to consider when investigating the heterogeneity between these studies. Adult felids are more likely to harbour *T. gondii* infection than younger age groups (Zhu et al., 2021). However, faecal shedding of oocysts, and thus the transmission of infection, occurs more in young felids, resulting in a complex dynamic that significantly impacts the prevalence of toxoplasmosis in any given population of wild or domestic felids (Zhu et al., 2021). Furthermore, study period can also influence these results, since infection rates are reported to be higher in autumn, winter and in rainy years (Hatam‐Nahavandi et al., 2021).

We also report a *T. gondii* prevalence of 48% among sheep in this region (Figure 4). This estimate is higher than those reported in other regions, such as Ethiopia and Iran (22.9% and 14.4% respectively), while still being lower than those reported in Sudan and Brazil (57.5% and 85% respectively) (Bahreh et al., 2021; Bekele & Kasali, 1989; Clementino et al., 2009; Medani & Kamil, 2014). It is worth mentioning that some of the aforementioned countries are examples of major exporters of sheep and livestock to countries in the Arabian Peninsula, which might explain the relatively high prevalence of *T. gondii* infection among this animal population in this region. Furthermore, some studies from Saudi Arabia and Kuwait examined ewes that went through abortion and linked that to their infection by *T. gondii*, as bradyzoites were commonly detected in aborted foetuses (Alazemi, 2014; Hussein et al., 2011). This outlines how toxoplasmosis can be an important cause of reproductive wastage in small ruminants, which may present a serious economic challenge to exporting countries (Hussein et al., 2011). Similar to our meta‐analysis of reports in felids, we also found severe heterogeneity in this subgroup. Although there is also an element of methodological diversity, as *T. gondii* infections were detected using multiple diagnostic techniques, we believe that clinical diversity is possibly a more important source of heterogeneity in this subgroup, as hygiene and breeding environments are very important determinants of infection rates in sheep (Alanazi, 2013). Moreover, different age groups were used as sample populations in different studies. Hotea et al. (2021) argued that the prevalence of toxoplasmosis increases with age, as older age groups are more likely to have been exposed to environmental contaminants at some point during their lives.

This review also identified some studies that reported *T. gondii* infection in other animal species thin this region, ranging from certain herbivores (e.g. goats and horses), to carnivores (e.g. striped hyenas and wolves), and omnivores (e.g. mongoose). We were unable to perform any statistical analysis on those prevalence estimates because of the low number of included studies per subgroup. However, a general observable trait in these reports was a higher prevalence in carnivores compared to herbivores. Although this difference can easily be attributed to the difference in feeding habits between these two subgroups, various reasons can also come into account such as the geographical location, age, gender of the animal, and the income level of the countries captivating these animals (Stelzer et al., 2019). Among the carnivores, wolves had the highest *T. gondii* prevalence. This is possibly due to the variation in their feeding habits, since these creatures live in different environmental conditions, which increases their risk of consuming *T. gondii* oocysts from multiple preys, especially sheep and goats (Figueiredo et al., 2020). On the other hand, horses and goats had higher *T. gondii* prevalence compared to camels, as camels usually live in arid regions that rarely allows them to get in direct contact with cats or ingest oocysts from contaminated water and food sources (Gebremedhin et al., 2016). Regarding omnivores, mongoose had the highest prevalence compared to other animals. This can be attributed to the feeding habits and lifestyle of mongoose, as they mostly feed on a variety of mammals which might be infected with *T. gondii* and acquire certain nutritional supplements from tunnels which increases their interaction with the possibly contaminated soil.

This systematic review and meta‐analysis attempt to provide a comprehensive summary and analysis of the prevalence of *T. gondii* in the Arabian Peninsula, a region where livestock is of utmost importance to the stability of local economies, and an infection that imposes a serious risk of reproductive wastage in this animal population. However, this review has some limitation, as the high levels of heterogeneity and the risk of publication bias that we estimate in our meta‐analysis can negatively impact the reliability of these prevalence estimates. Moreover, the failure to retrieve any studies from certain countries in the Arabian Peninsula (i.e. Bahrain, Oman and Yemen) can hinder the generalisability of our results to the entire region. Nevertheless, our findings provide valuable insight to local health authorities and policy makers who advocate a ‘One Health’ approach to reduce the impact of this deleterious infection on both humans and animals. They also highlight the need for further, more extensive, well‐designed studies to investigate the epidemiology of toxoplasmosis in various disease transmission settings including the environment (e.g. water, soil, and domestic and wild animals) and in humans (e.g. healthy and immuno‐compromised people, pregnant women and children) to better understand the burden and the transmission dynamics of the parasite in this region. Furthermore, it calls for more efforts to be directed towards infection control and prevention such as health education, provision of clean drinking water for animals, and proper cooking of meat. It is also plausible to suggest limiting the numbers of cats around farm animals, providing troughs for feed and water, and investigating the possible use of available vaccines to reduce *T. gondii* infection in domestic animals.

## AUTHOR CONTRIBUTIONS

Asmaa Abdelgadier, Nada Assaad and Zaynab Elhussein have contributed equally to this manuscript and are co‐first authors. Data curation and writing – original draft: Asmaa Abdelgadier, Nada Assaad, Zaynab Elhussein and Sami Suliman. Project administration: Abdulla M. Al‐Marri. Data curation, formal analysis, methodology, software, validation, visualisation, writing – original draft and writing – review & editing: Khalid Eltom. Project administration and writing – review & editing: Ebtisam A. Al‐Mslemani and Abdul Azia Al‐Zeyara. Writing – original draft and writing – review & editing: Abdel Rahim El Hussein. Conceptualisation, project administration, supervision, and writing – review & editing: Khalid A. Enan.

## FUNDING

The authors did not receive any funding for the purpose of this review article.

## CONFLICT OF INTEREST

The authors declare no conflict of interest.

## ETHICAL STATEMENT

The authors confirm that the ethical policies of the journal, as noted on the journal's author guidelines page, have been adhered to. No ethical approval was required as this is a review article with no original research data.

### PEER REVIEW

The peer review history for this article is available at https://publons.com/publon/10.1002/vms3.1004.

## Supporting information

supplementary Information

supplementary Information

supplementary Information

supplementary Information

supplementary Information

supplementary Information

supplementary Information

## Data Availability

The data that support the findings of this study are available from the corresponding author upon reasonable request.
