# Supplementary material for: Prevalence of Toxoplasma gondii infection in animals of the Arabian Peninsula between 2000–2020: A systematic review and meta‐analysis
Source: Vet Med Sci. 2022 Nov 21;9(1):471–80. doi: 10.1002/vms3.1004 (PMC9857116; doi:10.1002/vms3.1004)
Supplement: Supplementary file 2 — supplementary Information [file VMS3-9-471-s005.docx]

**Search strategy in PubMed**

((“toxoplasmosis”[Title/Abstract]) OR (“toxoplasma”[Title/Abstract]) OR (“T. gondii”[Title/Abstract])) AND ((“Qatar”[Title/Abstract]) OR (“Saudi Arabia”[Title/Abstract]) OR (“United Arab Emirates”[Title/Abstract]) OR (“KSA”[Title/Abstract]) OR (“UAE”[Title/Abstract]) OR (“Bahrain”[Title/Abstract]) OR (“Yemen”[Title/Abstract]) OR (“Kuwait”[Title/Abstract]) OR (“Oman”[Title/Abstract]))

**Search strategy in ScienceDirect**

**Advanced search ---> title, abstract or author-specified keywords**

((“toxoplasma”) OR (“toxoplasmosis”)) AND ((“Qatar”) OR (“Saudi Arabia”) OR (“United Arab Emirates”) OR (“KSA”) OR (“UAE”))

((“toxoplasma”) OR (“toxoplasmosis”)) AND ((“Bahrain”) OR (“Yemen”) OR (“Kuwait”) OR (“Oman”))

**Search strategy in Cochrane library**

((“toxoplasma”) OR (“toxoplasmosis”)) AND ((“Qatar”) OR (“Saudi Arabia”) OR (“United Arab Emirates”) OR (“KSA”) OR (“UAE”) OR (“Bahrain”) OR (“Yemen”) OR (“Kuwait”) OR (“Oman”))

**Search strategy in Google scholar**

**Advanced search ---> with the exact phrase**

In the title of the article

("toxoplasma" OR "toxoplasmosis") AND (“Qatar” OR “Saudi Arabia” OR “United Arab Emirates” OR “KSA” OR “UAE” OR “Bahrain” OR “Yemen” OR “Kuwait” OR “Oman”)

**Search strategy in Scopus**

**Search within ---> Article title, Abstract, Keywords**

("toxoplasma" OR "toxoplasmosis") AND (“Qatar” OR “Saudi Arabia” OR “United Arab Emirates” OR “KSA” OR “UAE” OR “Bahrain” OR “Yemen” OR “Kuwait” OR “Oman”)
