# Supplementary material for: Prevalence of Toxoplasma gondii infection in animals of the Arabian Peninsula between 2000–2020: A systematic review and meta‐analysis
Source: Vet Med Sci. 2022 Nov 21;9(1):471–80. doi: 10.1002/vms3.1004 (PMC9857116; doi:10.1002/vms3.1004)
Supplement: Supplementary file 3 — supplementary Information [file VMS3-9-471-s003.docx]

**Supplementary file:** Animal species which were found to harbour Toxoplasma gondii infections in the Arabian Peninsula.

| **Common name** | **Current taxonomy** |
| --- | --- |
| Felids | **Family:** Felidae  *Caracal caracal* (United Arab Emirates), *Felis catus* (United Arab Emirates), *Felis margarita harrisoni* (Qatar), *Acinonyx jubatus soemmeringii* (United Arab Emirates, Qatar), *Felis silvestris gordoni* (Qatar, United Arab Emirates), *Panthera pardus nimr* (United Arab Emirates), *Acinonyx jubatus rex* (Qatar) |
| Camels | **Family:** Camelidae  Camelus dromedarius ( Saudi Arabia) |
| Fox, Jackal and Wolf | **Family:** Canidae  *Vulpes cana* (United Arab Emirates*), Vulpes rueppellii* (United Arab Emirates), *Vulpes vulpes arabica (*United Arab Emirates), *Canis aureus* (United Arab Emirates*), Canis lupus arabs* (United Arab Emirates) |
| Hedgehogs | **Family:** Erinaceidae  *Paraechinus hypomelas* (United Arab Emirates), *Hemiechinus auritus* (United Arab Emirates), *Paraechinus aethiopicus* (United Arab Emirates) |
| Mongoose | **Family:** Herpestidae  *Urva edwardsii* (United Arab Emirates), *Ichneumia albicauda* (United Arab Emirates) |
| Hyena | **Family:** Hyaenidae  *Hyaena hyaena* (United Arab Emirates) |
| Rats | **Family:** Muridae  *Rattus rattus* (Saudi Arabia) |
| Others (sheep, horses, chickens, and goats) | Unspecified species |
