# Supplementary material for: Prevalence of Toxoplasma gondii infection in animals of the Arabian Peninsula between 2000–2020: A systematic review and meta‐analysis
Source: Vet Med Sci. 2022 Nov 21;9(1):471–80. doi: 10.1002/vms3.1004 (PMC9857116; doi:10.1002/vms3.1004)
Supplement: Supplementary file 4 — supplementary Information [file VMS3-9-471-s006.docx]

**Supplementary file**. Quality assessment (critical appraisal) of included studies using the Joanna Briggs Institute (JBI) critical appraisal tool for prevalence studies.

|  | *Was the sample frame appropriate to address the target population?** | *Were study participants sampled in an appropriate way?* | *Was the sample size adequate?* **^‡^** | *Were the study subjects and setting described in detail?* | *Was the data analysis conducted with sufficient coverage of the identified sample?* | *Were valid methods used for the identification of the condition?* | *Was the condition measured in a standard, reliable way for all participants?* | *Was there appropriate statistical analysis?* | *Was the response rate adequate, and if not, was the low response rate managed appropriately?* | Total  Score |
| --- | --- | --- | --- | --- | --- | --- | --- | --- | --- | --- |
| Al Naser | u/c | N/A | u/c | Yes | Yes | u/c | Yes | Yes | Yes | 5 |
| Alanazi | u/c | N/A | u/c | Yes | Yes | Yes | Yes | Yes | Yes | 6 |
| Alanazi | u/c | N/A | u/c | Yes | u/c | Yes | Yes | Yes | u/c | 4 |
| Al-anazi | u/c | N/A | u/c | Yes | Yes | Yes | Yes | Yes | Yes | 6 |
| Alazemi | u/c | N/A | u/c | Yes | Yes | Yes | Yes | Yes | Yes | 6 |
| Almogren | u/c | u/c | u/c | Yes | u/c | Yes | Yes | u/c | u/c | 3 |
| Al-Mulhim | u/c | u/c | u/c | Yes | u/c | u/c | Yes | Yes | u/c | 3 |
| Boughattas | u/c | N/A | u/c | Yes | u/c | Yes | Yes | Yes | u/c | 4 |
| Dubey | u/c | N/A | u/c | Yes | u/c | Yes | Yes | Yes | u/c | 4 |
| Dubey | u/c | N/A | u/c | Yes | u/c | Yes | Yes | Yes | u/c | 4 |
| Elamin | u/c | N/A | u/c | No | Yes | u/c | Yes | Yes | Yes | 4 |
| Elamin | u/c | N/A | u/c | Yes | u/c | u/c | Yes | Yes | u/c | 3 |
| El-azay | Yes | N/A | Yes | Yes | Yes | u/c | Yes | Yes | Yes | 7 |
| Hussein | u/c | N/A | u/c | Yes | Yes | Yes | Yes | Yes | Yes | 6 |
| Hussein | u/c | N/A | u/c | Yes | u/c | Yes | Yes | Yes | u/c | 4 |
| Ismael | u/c | N/A | u/c | Yes | u/c | Yes | Yes | Yes | u/c | 4 |
| Mohamed | u/c | N/A | u/c | Yes | u/c | Yes | Yes | Yes | u/c | 4 |
| Mohamed | u/c | N/A | u/c | Yes | u/c | Yes | Yes | Yes | u/c | 4 |
| Schuster | u/c | N/A | u/c | Yes | u/c | u/c | No | Yes | u/c | 2 |
