# Supplementary figures and images for: Prevalence of Toxoplasma gondii infection in animals of the Arabian Peninsula between 2000–2020: A systematic review and meta‐analysis
Source: Vet Med Sci. 2022 Nov 21;9(1):471–80. doi: 10.1002/vms3.1004 (PMC9857116; doi:10.1002/vms3.1004)

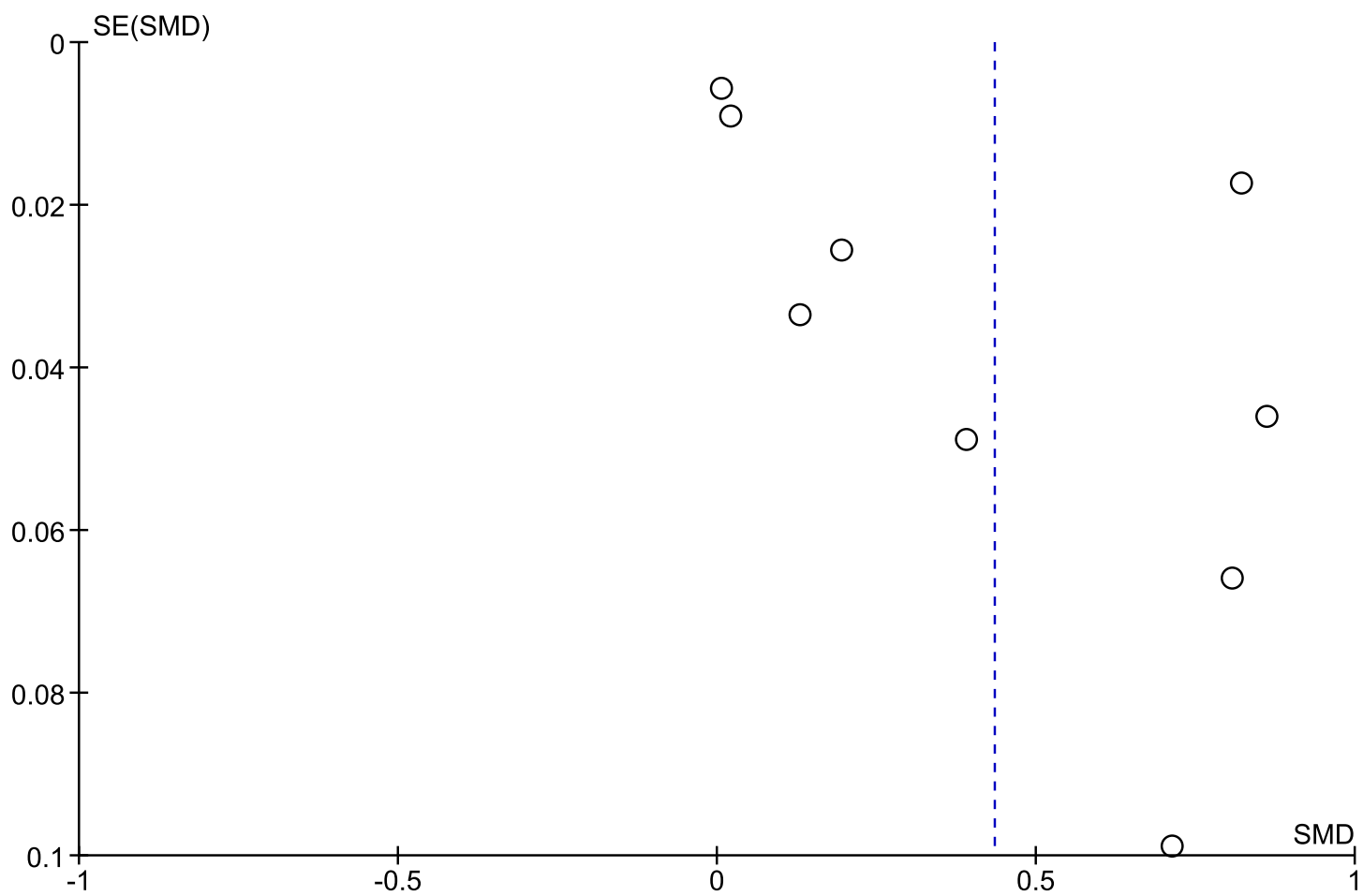

Supplement: Supplementary file 5 — supplementary Information [file VMS3-9-471-s002.pdf]

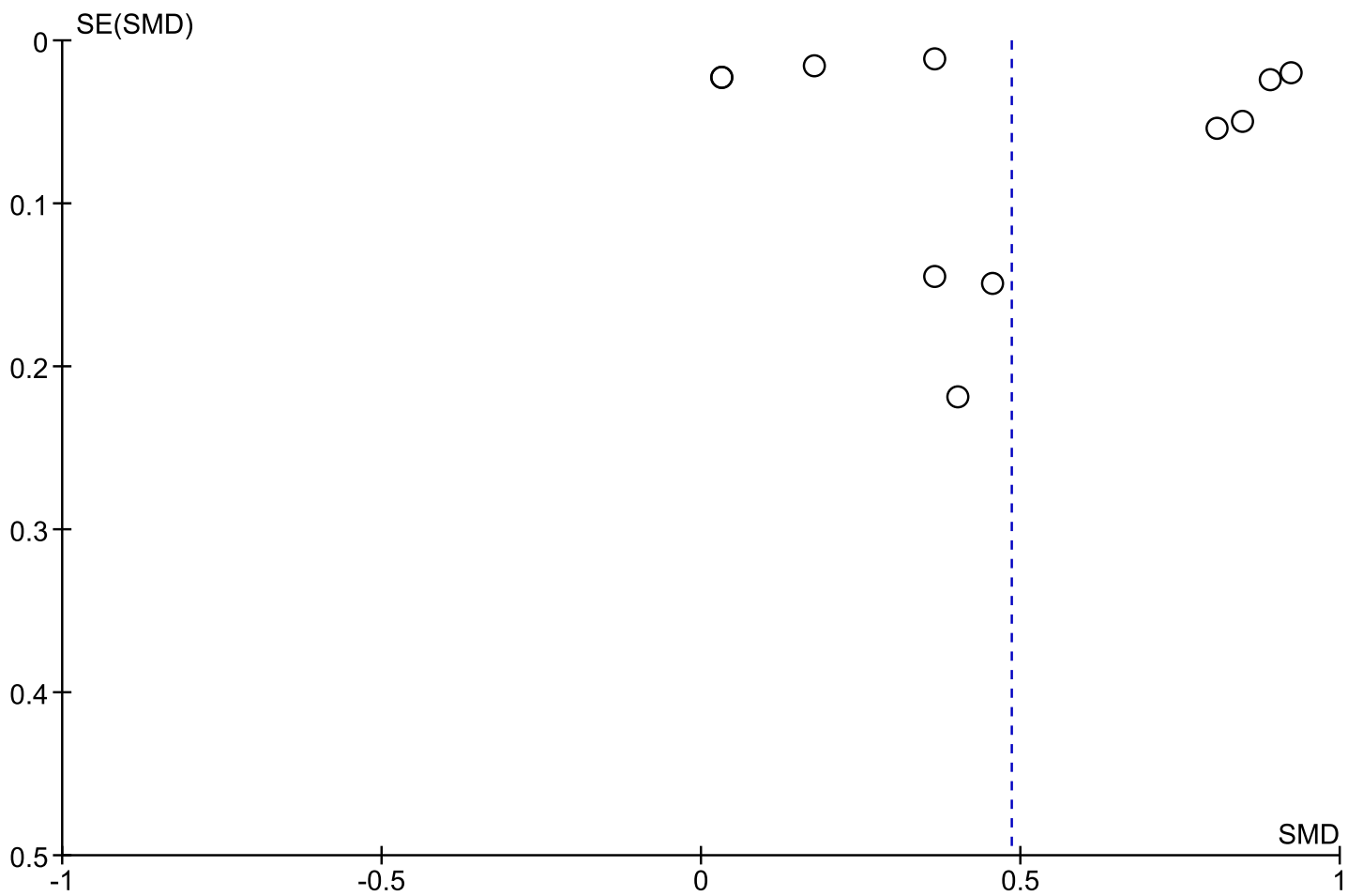

Supplement: Supplementary file 6 — supplementary Information [file VMS3-9-471-s001.pdf]

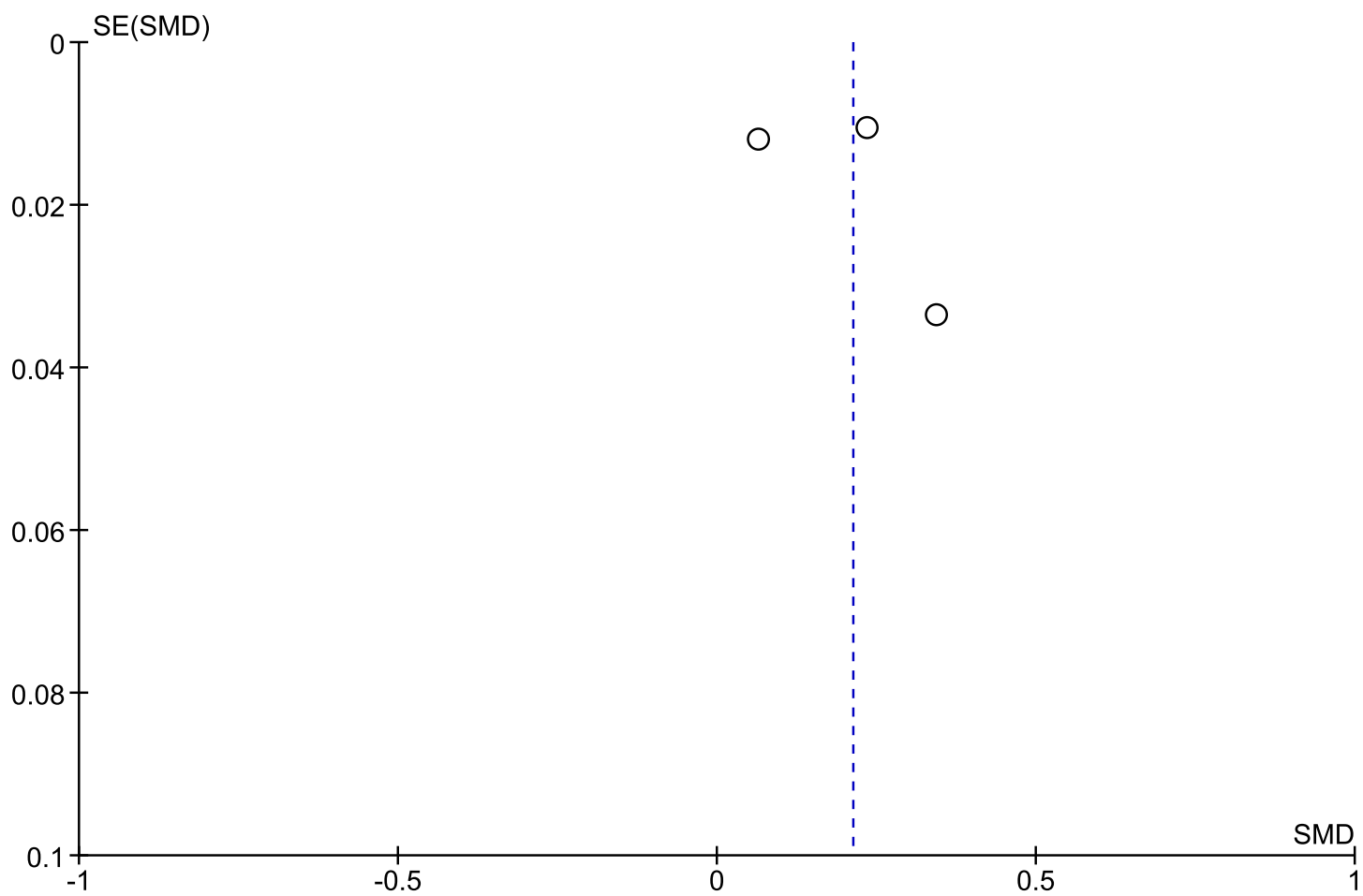

Supplement: Supplementary file 7 — supplementary Information [file VMS3-9-471-s004.pdf]
